# Negative and positive externalities in intergroup conflict: exposure to the opportunity to help the outgroup reduces the inclination to harm it

**DOI:** 10.3389/fpsyg.2015.01594

**Published:** 2015-10-31

**Authors:** Ori Weisel

**Affiliations:** Centre for Decision Research and Experimental Economics, University of NottinghamNottingham, UK

**Keywords:** parochialism, intergroup conflict, ingroup love, outgroup hate, team games

## Abstract

Outgroup hate, in the context of intergroup conflict, can be expressed by harming the outgroup, but also by denying it help. Previous work established that this distinction—whether the externality on the outgroup is negative or positive—has an important effect on the likelihood of outgroup hate emerging as a motivation for individual participation in intergroup conflict. The current work uses a within-subject design to examine the behavior of the same individuals in intergroup conflict with negative and positive externalities on the outgroup. Each participant made two choices, one for each type of externality, and the order was counter balanced. The main results are that (1) behavior is fairly consistent across negative and positive externalities, i.e., the tendency to display outgroup hate by harming the outgroup is correlated with the tendency to display outgroup hate by avoiding to help the outgroup; (2) People are reluctant to harm the outgroup after being exposed to the opportunity to help it; (3) *Groupness*—the degree to which people care about their group and its well-being—is related to outgroup hate only when participants encounter the opportunity to harm the outgroup first (before they encounter the opportunity to help it). In this setting the relationship between groupness and outgroup hate spilled over to the subsequent interaction, where it was possible to help the outgroup. When the opportunity to help the outgroup was encountered first, groupness was not related to outgroup hate.

## 1. Introduction

In the context of intergroup conflict, parochial altruism—the willingness to incur a personal cost in order to favor one's ingroup over the outgroup—can be motivated by “ingroup love” (a cooperative preference for helping the ingroup) and/or by “outgroup hate” (an aggressive/competitive preference for harming the outgroup, or increasing the gap between the groups; Rusch, 2014). In many intergroup conflicts the two are not distinguishable, as individual participation in the conflict simultaneously increases the ingroup's welfare and decreases the outgroup's welfare, such that participation can be motivated by ingroup love, by outgroup hate, or by a combination of both (Allport, 1954; Bornstein and Ben-Yossef, 1994; Brewer, 1999).

Outgroup hate is often thought of as a desire to actively harm the outgroup, e.g., by taking part in hate crimes or property destruction targeted against the outgroup. In such cases, outgroup hate is expressed by imposing negative externalities on members of the outgroup. Outgroup hate can also manifest itself, however, as discriminatory helping behavior. In the latter case, outgroup hate is expressed by avoiding to help, or, in other words, avoiding to impose positive externalities, on members of the outgroup. In a recent paper, Weisel and Böhm (2015) show that the relative roles of ingroup love and outgroup hate as motivations for individual participation in intergroup conflict crucially depend on whether outgroup hate can be expressed by imposing negative externalities, or by avoiding to impose positive externalities, on the outgroup.

Outgroup hate emerges as an important motivation for individual participation in intergroup conflict when it can be displayed by help-avoidance (avoiding to impose a positive externality), especially when the degree of enmity between the groups is high (Weisel and Böhm, 2015). When outgroup hate necessarily entails harming the outgroup (i.e., imposing a negative externality), it plays a lesser role, and ingroup love seems to be the main motivation at play (Halevy et al., 2008, 2012; De Dreu, 2010; De Dreu et al., 2010). The above result—that outgroup hate plays a major role when it can be expressed by help-avoidance—is in line with previous work that finds that many instances of discrimination are driven by *ingroup favoritism* (the selective preferential treatment of ingroup members; sometimes used synonymously to *ingroup love*), and not necessarily by *outgroup hostility* (outright outgroup derogation; sometimes used synonymously to (*outgroup hate*; Mummendey et al., 1992, 2000; Banaji and Greenwald, 2013; Greenwald and Pettigrew, 2014).

Weisel and Böhm (2015) had participants make decisions in the context of three experimentally induced intergroup conflicts, namely the Intergroup Prisoner's Dilemma (IPD; Bornstein and Ben-Yossef, 1994), the Intergroup Prisoner's Dilemma—Maximizing Difference game (IPD-MD; Halevy et al., 2008), and a positive variant of the IPD-MD (introduced by Weisel and Böhm), in a between-subjects design. The present study employs a within-subjects design and focuses on the IPD-MD and the positive variant of the IPD-MD. In the IPD-MD game participants face a choice between selfish behavior, helping their ingroup (at a personal cost), or helping their ingroup *and* harming the outgroup (at the same personal cost; see Table 1). In the positive variant of the IPD-MD the choice is between selfish behavior, helping the ingroup (at a personal cost), or helping the ingroup and *helping* the outgroup as well (at the same cost; see Table 1).

**Table 1:** **Games, accounts, and payoffs**.

**Group size = 3**		**Effect on**											
		**Ingroup**						**Outgroup**					
**Game**	**Account**	**Self**		**2**		**3**		**1**		**2**		**3**	
IPD-MD	Private	+2		0		0		0		0		0	
	Within-group	+1		+1		+1		0		0		0	
	Between-group	+1		+1		+1		−1		−1		−1	
Positive variant of the IPD-MD	Private	+2		0		0		0		0		0	
	Within-group	+1		+1		+1		0		0		0	
	Between-group	+1		+1		+1		+1		+1		+1	
**Group size** = **6**		**Effect on**											
		**Ingroup**						**Outgroup**					
**Game**	**Account**	**Self**	**2**	**3**	**4**	**5**	**6**	**1**	**2**	**3**	**4**	**5**	**6**
IPD-MD	Private	+2	0	0	0	0	0	0	0	0	0	0	0
	Within-group	+0.5	+0.5	+0.5	+0.5	+0.5	+0.5	0	0	0	0	0	0
	Between-group	+0.5	+0.5	+0.5	+0.5	+0.5	+0.5	−0.5	−0.5	−0.5	−0.5	−0.5	−0.5
Positive variant of the IPD-MD	Private	+2	0	0	0	0	0	0	0	0	0	0	0
	Within-group	+0.5	+0.5	+0.5	+0.5	+0.5	+0.5	0	0	0	0	0	0
	Between-group	+0.5	+0.5	+0.5	+0.5	+0.5	+0.5	+0.5	+0.5	+0.5	+0.5	+0.5	+0.5

*The table illustrates the effect of each token allocated to the private, within-group, or between-group accounts on the payoff of the individual making the allocation (referred to as ingroup member “self”), the payoff of each of the two other ingroup members, and the payoff of the three outgroup members. Each individual had 10 tokens to allocate. The final payoff of each person was determined by the combined effect of the allocations of all ingroup and outgroup members*.

The logic underlying the analysis in Weisel and Böhm (2015) is that the IPD-MD and the positive variant of the IPD-MD are useful tools for investigating ingroup love and outgroup hate because the decisions made in the context of these games make the two key motivations—ingroup love and outgroup hate—distinguishable from each other. As stated above, helping the ingroup in the IPD-MD can be achieved either with or without harming the outgroup. As a result, choosing to help the ingroup while harming the outgroup was interpreted by Weisel and Böhm (2015) as a display of outgroup hate, and choosing to help the ingroup without harming the outgroup was interpreted as a display of ingroup love (an interpretation shared by, e.g., Halevy et al., 2008, 2012; De Dreu et al., 2010). In a similar vein, helping the ingroup in the positive variant of the IPD-MD can be achieved either with or without helping the outgroup. Crucially to the analysis in Weisel and Böhm, choosing to help the ingroup without helping the outgroup was interpreted as a display of outgroup hate, and choosing to help the ingroup and helping the outgroup as well was interpreted as a display of ingroup love^1^.

An initial goal of the present study is to examine whether choices in the IPD-MD and in the positive variant of the IPD-MD indeed have comparable motivational underpinnings. Weisel and Böhm interpret (1) harming the outgroup in the IPD-MD, and avoiding to help the outgroup in the positive variant of the IPD-MD, as manifestations of outgroup hate; and (2) avoiding to harm the outgroup in the IPD-MD, and helping the ingroup and the outgroup in the positive variant of the IPD-MD, as manifestations of ingroup love. To the degree that these interpretations are reasonable, people who choose to help the ingroup and harm the outgroup in the IPD-MD should also show a preference for helping the ingroup and avoiding to help the outgroup in the positive variant, as both of these actions are, supposedly, displays of outgroup hate; and people who choose to help the ingroup without harming the outgroup in the IPD-MD should also show a preference for helping the ingroup and the outgroup in the positive variant, as both actions are, supposedly, related to ingroup love.

An additional question that the current study addresses is that of order effects. Does the order in which people encounter situations in which they can display outgroup hate by imposing negative externalities on the outgroup, and situations in which they can display outgroup hate by avoiding to impose positive externalities on the outgroup, affect behavior? Past work suggests, albeit indirectly, that exposure to the possibility of harming the outgroup might lead to more negative attitudes toward it (i.e., more outgroup hate) in future interactions, and that the possibility to help the outgroup might lead to more positive attitudes toward it in the future. This prediction is derived from work showing that perceptions of the outgroup (in particular dehumanization and rehumanization) are affected by awareness of harm or help that members of the ingroup imposed on the outgroup in the past (Castano and Giner-Sorolla, 2006; Čehajić et al., 2009; Saguy et al., 2015). The present design allows to examine whether exposure to the opportunity to harm or to help the outgroup, and the understanding that other ingroup member have the same opportunity, has a similar effect on future behavior. The results can have implications on the way repeated interactions between groups are structured, as well as methodological implications for research using the IPD-MD and related paradigms.

Finally, the study addresses the relation between peoples' sense of groupness—defined as the degree to which they care about their group and its well-being—and their willingness to display outgroup hate by imposing negative externalities, and/or by avoiding to impose positive externalities, on the outgroup. As groupness is measured by items that concern the ingroup only (see Section 2.3.1), a straight forward prediction is that it is related to the overall willingness to contribute to the ingroup, regardless of the effect on the outgroup. The respective relations between groupness and ingroup love and outgroup hate are less obvious to predict. Would groupness be related to ingroup love, to outgroup hate, or to both? Results from studies investigating the hormone oxytocin, known for increasing peoples' affinity to their group (i.e., their groupness), suggest that it is associated mainly with ingroup love, as opposed to outgroup hate (De Dreu et al., 2010; De Dreu, 2012).

The groupness measure that is introduced here can be seen as a type of social value orientation (SVO) measure that is targeted at one's ingroup. Typically, SVO is conceptualized as concerning an unidentified other, not necessarily a member of the in-group (see Section 2.3.3). The groupness measure used here (Section 2.3.1) explicitly focuses on the well-being of the ingroup. Despite this difference, it would hardly be surprising if the two measures turn out to be very related to each other; indeed, recent work suggests that pro-social tendencies are often ingroup-bounded (De Dreu et al., 2014, 2015). Although both groupness and SVO are measured in the current experiment, the main interest here is the possible relation of groupness—a concern for the well-being of one's ingroup—to displays of outgroup hate vis-à-vis an outgroup.

## 2. Materials and methods

### 2.1. Participants

One hundred forty-four undergraduate students (74 females, *M*_*age*_ = 25) at the Hebrew University of Jerusalem participated in the experiment, which was approved by the psychology department's ethics committee. Participants were recruited by campus advertisements promising monetary rewards for participation in a decision-making task.

### 2.2. Experimental procedure

Sessions were held with cohorts of twelve participants. Upon arrival each participant was seated in a separate cubicle, and given printed instructions and decision forms (see Supplementary Material).

### 2.3. Design

The independent variables were the game (IPD-MD vs. positive variant of the IPD-MD; within-subjects) and the size of the interacting groups (three vs. six; between-subjects). The order of the two games was counter-balanced across sessions. The two orders of the game and the two group sizes were perfectly balanced, resulting in 36 participants in each cell. The group size did not affect the results in any meaningful way, so this variable was dropped from the analysis, and the observations were pooled, resulting in 72 participants in each order of the games.

The twelve participants in each session were randomly divided into groups of size three or six. Each group was matched with another group. Groups were named, and graphically represented on the instructions, as the *circles* group, the *triangles* group, the *diamonds* group, and the *squares* groups (when the group size equalled six, there were only two groups: circles and triangles). Participants were informed that they will be required to make decisions in two *world states*, color coded as the yellow and green world states; that at the end of the experiment one of the two world states will be chosen by a public coin toss; and that decisions made in the chosen state will determine the payoffs of all participants. No feedback was given between decisions in the two world states. To increase the saliency of the distinction between the two world states, the instructions that explained the yellow world state were printed on yellow paper, as were the corresponding decision forms. Likewise, the instructions that explained the green world state, and the corresponding decision forms, were printed on green paper.

In both the green and the yellow world states each participant was endowed with ten tokens, and had to allocate them between a private account, a within-group account, and a between-group account. Participants decided on one allocation of tokens in each world state. In the green state they played the IPD-MD game, and in the yellow state the positive variant of the IPD-MD game. The payoffs associated with each account in each of these games, and for each of the two group sizes, are displayed in Table 1 (see Supplementary Material for the instructions participants received, and for formal payoff functions).

It is apparent from Table 1 (see also the payoff functions in the Supplementary Material) that in both the IPD-MD and the positive variant, regardless of group size, payoff maximizing players should invest all of their tokens in the private account, which generates a profit of 2 New Israeli Shekels (NIS) for the decision maker. Investing in the other accounts generates only 1 (group size = 3) or 0.5 (group size = 6) NIS. This is the case regardless of the actions of the other ingroup and/or outgroup members. Since no feedback was provided between the two games, they can be thought of as two separate one-shot games, and the Nash equilibrium is to invest all of the tokens in the private account in both the IPD-MD and the positive variant.

In the IPD-MD negative payoffs are possible. The worst-case scenario for a given individual is to allocate all of her tokens to the within- or between-group account, which generates a profit of either 10 NIS (group size = 3) or 5 NIS (group size = 6) for herself, while her group members keep all of their tokens in their private account, which does not affect the individual's payoff, and all outgroup members allocate all of their tokens to the between-group account, which leads to a loss of 30 NIS for the individual. In this case the individual's payoff is −20 or −25 NIS (depending on the group size). To avoid negative payoffs, an initial amount of 40 NIS was added to each participant's total payoff in the IPD-MD, ensuring a minimum payoff of 20 or 15 NIS. In the positive variant of the IPD-MD an initial amount of 10 NIS was added to each participant, such that the minimum payoff is 20 or 15 NIS as well.

A post experimental questionnaire tapped participants' sense of “groupness,” i.e., the degree to which they cared for and identified with their group; their social value orientation; as well as beliefs about the allocations of ingroup and outgroup members (the latter are not reported in the current work).

#### 2.3.1. Groupness

The following four items, rated on a 1 (“do not agree at all”) to 7 (“totally agree”) scale, captured participants' sense of groupness:

1. It is important to me to contribute to the group.
2. I am committed to contribute to the group.
3. It is important to me to act in favor of the group.
4. I want the group to do well.

#### 2.3.2. Beliefs

Participants were asked to best estimate the average number of tokens that the other members in their group and in the other group chose to keep and to invest in each account in both the green (IPD-MD) and yellow (positive variant) world states.

#### 2.3.3. Social value orientation

Participants' social value orientation (SVO)—the way they balance between their own and others' welfare—was assessed with the social value orientation decomposed game measure (Van Lange, 1999). The measure is based on nine items. In each item participants choose one of three allocations of resources between themselves and an anonymous other person. One of the three allocations indicates a pro-social preference to maximize the joint outcome of self and other, another indicates an individualistic preference to maximize the outcome of self, and the third indicates a competitive preference to maximize the gap between self and other. An example is the choice between 500 points to self and 100 to other (the competitive option), 500 points to self and 500 points to other (the pro-social option), or 550 points to self and 350 to other (the individualistic option). Participants who make at least six choices that are consistent with one of the three types are classified as that type (e.g., a participant who makes six (or more) pro-social choices is classified as pro-social).

## 3. Results

### 3.1. Consistency between IPD-MD and the positive variant of the IPD-MD

Are the motivations associated with contribution to the within-group and between-group accounts in the IPD-MD—ingroup love and outgroup hate, respectively—indeed a mirror image of the motivations in the positive variant of the IPD-MD? In other words, are people who contribute to the within-group (between-group) account in the IPD-MD more likely to contribute to the between-group (within-group) account in the positive variant? According to Weisel and Böhm (2015), this should indeed be the case.

Table 2 presents correlations between the number of tokens each participant allocated to the within- and between-group accounts in the IPD-MD and the positive variant of the IPD-MD. Regardless of which game was played first, the correlation between allocations to the within-group account in the IPD-MD and the between-group account in the positive variant (*r* = 0.64, *r* = 0.45) was medium-high and significantly different from zero. The same holds true for the correlation between the between-group account in the IPD-MD and the within-group account in the positive variant (*r* = 0.48, *r* = 0.72). In contrast, correlations between the within-group accounts (*r* = −0.15, *r* = −0.10), and between the between-group accounts (*r* = −0.11, *r* = −0.01), are low and not significantly different from zero. This pattern of results confirms that the motivations underlying contribution to the within-group and between-group accounts in the IPD-MD (ingroup love and outgroup hate, respectively) are indeed a mirror image of the motivations in the positive variant of the IPD-MD, as argued in Weisel and Böhm (2015).

**Table 2:** **Correlations between contribution decisions in the first and second games**.

			**2nd game**			
			**IPD-MD**		**Positive variant of the IPD-MD**	
			**Within-group**	**Between-group**	**Within-group**	**Between-group**
**1st game**	**IPD-MD**	**Within-group**	−	−	−0.15	0.64^***^
		**Between-group**	−	−	0.48^***^	−0.11
	**Positive variant of the IPD-MD**	**Within-group**	−0.10	0.72^***^	−	−
		**Between-group**	0.45^***^	−0.01	−	−

*** *p < 0.001*.

### 3.2. Order effects

**Figure 1:**
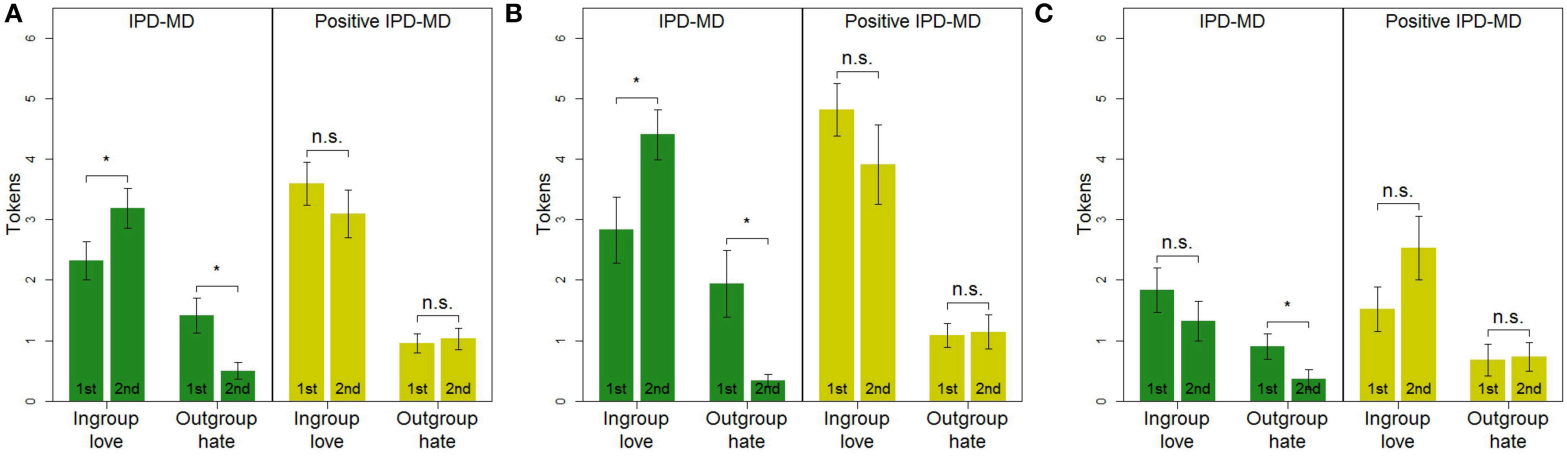
**Allocations to ingroup love and outgroup hate in the IPD-MD and Positive IPD-MD**. **(B,C)** Are restricted to either pro-social or individualistic participants, respectively. Each pair of bars refers to conditions where the respective game was played first (left) or second (right). Ingroup love stands for the within-group account in the IPD-MD and for the between-group account in the positive variant of the IPD-MD; outgroup hate stands for the between-group account in the IPD-MD, and for the within-group account in the positive variant. ^*^p < 0.05; Wilcoxon Rank Sum test. **(A)** All participants (*n* = 144). **(B)** Pro-Social (*n* = 79). **(C)** Individualistic (*n* = 55).

Does behavior in a given game depend on whether it is played first or second? Figure 1A shows the average contributions toward ingroup love and outgroup hate, for each game and for each position the game was played (first or second). As can be seen in the figure, the order makes a difference only for the IPD-MD, but not for the positive variant of the IPD-MD. In the IPD-MD there was more outgroup hate, and less ingroup love, when it was played first, as compared to when it was played second (i.e., after the positive variant; Wilcoxon Rank Sum test: ingroup love, *p* = 0.029; outgroup hate, *p* = 0.002). In the positive variant of the IPD-MD allocations to both ingroup love and outgroup hate remained similar, regardless of whether it was played first or after the IPD-MD (ingroup love, *p* = 0.135; outgroup hate, *p* = 0.996).

A plausible way to interpret this finding is that the positive variant of the IPD-MD has a spillover effect on behavior in the IPD-MD, but not vice-versa. After having the option to help the outgroup in the positive variant of the IPD-MD, very few group members find it appropriate to harm the outgroup in a subsequent IPD-MD. The opposite is not true: having the option to harm the outgroup in the IPD-MD does not affect behavior in the positive variant that follows.

### 3.3. Social value orientation

Of the 144 participants that took part in the study, 79 (55%) were classified as pro-social, 55 (38%) as individualistic, 1 (1%) as competitive, and 9 (6%) were unclassified (see Table 3). Figures 1B,C show the average contributions toward ingroup love and outgroup hate for pro-social and individualistic participants, respectively. The figures show that the order effects reported in the previous section are driven by pro-social participants, while behavior of individualistic participants did not follow the same pattern.

**Table 3:** **Distribution of participants accross SVO and groupness levels**.

	**Groupness level**			
**SVO**	**High**	**Low**	**= Median**	**Total**
Pro social	49	29	1	79
Individualistic	16	35	4	55
Competitive	0	1	0	1
Unclassified	3	5	1	9
Total	68	70	6	144

*SVO was determined according to the SVO decomposed game measure. The High and Low groupness levels were determined according to a median split of the groupness scores*.

Previous work links pro-sociality to increased discriminatory behavior (in favor of the ingroup) in intergroup setting (Aaldering et al., 2013). In the present study, discriminatory behavior is manifested in the clearest way by contributions to the within-group account in the positive variant of the IPD-MD (i.e., outgroup hate). These choices are clearly discriminatory, since the same outcome for the ingroup can be achieved by investing in the non-discriminatory between-group account, which benefits the outgroup as well^2^. Pro-socials indeed invested more than individualistic participants in the discriminatory within-group account in the positive variant (Pro-social: *n* = 79, *M* = 1.11, *SD* = 1.47; Individualistic: *n* = 55, *M* = 0.71, *SD* = 1.30; Wilcoxon Rank Sum test: *p* = 0.054).

Social value orientation was highly related to the groupness measure. Pro-socials were clearly higher on groupness (*M* = 4.78, *SD* = 1.38) than individualistic participants [*M* = 3.49, *SD* = 1.61; *t*_(132)_ = 4.96, *p* < 0.001]. Despite this strong relation between SVO and groupness, the groupness score of a considerable number of participants is not in line with their SVO type. The groupness score of thirty-seven percent of the pro-social participants was below the median, and the groupness score of 29% of the individualistic participants was above the median (see Table 3), suggesting that while SVO and groupness are strongly related, they do not fully overlap.

### 3.4. Effect of groupness

The effect of groupness on contribution decisions was tested by means of generalized linear mixed effect models, using the lme4 package (Bates et al., 2012) in the R environment (R Core Team, 2012). Since each participant made two decisions, the specific participant was modeled as a random effect (Pinheiro and Bates, 2000). The explanatory variables were groupness, the game [dummy variable; IPD-MD (baseline) or positive variant of the IPD-MD], the position of the game [dummy variable; first (baseline) or second], and the two- and three-way interactions between these variables. The dependant variables were (in separate models) the number of tokens invested in the private account, the number of tokens invested toward ingroup love (within-group account in IPD-MD, between-group account in positive variant), and the number of tokens invested toward outgroup hate (between-group account in IPD-MD, within-group account in positive variant).

Table 4 presents the results of three regression models, predicting the number of tokens invested in the private account, invested toward ingroup love, and invested toward outgroup hate, as a function of the participants reported level of groupness, the game, and the position of the game (first or second). To facilitate interpretation, the table reports the intercept and slope for the groupness variable for each combination of game and position, rather than the effect of each variable relative to a baseline. Accordingly, the significance indicators in Table 4 refer to comparisons of the intercepts and slopes to zero, rather than to an (arbitrary) baseline (see Supplementary Material for another presentation of these results).

**Table 4:** **Generalized linear mixed effects model**.

**Game**	**Position**	**Private account**		**Ingroup love**		**Outgroup hate**	
		**Intercept**	**Slope**	**Intercept**	**Slope**	**Intercept**	**Slope**
IPD-MD	Fisrt	11.21^***^	−1.19^***^	−0.83	0.76^***^	−0.38	0.43^**^
Positive variant of the IPD-MD	Second	10.51^***^	−1.11^***^	−0.53	0.87^***^	0.02	0.24^†^
Positive variant of the IPD-MD	First	11.04^***^	−1.30^***^	−1.29	1.13^***^	0.25	0.17
IPD-MD	Second	11.35^***^	−1.17^***^	−1.50^†^	1.09^***^	0.14	0.08

*The number of tokens invested the private account, ingroup love, and outgroup hate (in separate models), as a function of groupness, the game (dummy variable with two levels: IPD-MD or the positive variant), whether the game was played first or second (dummy variable with two levels), and the interactions between these three variables. The intercept and slope of groupness for each level of the dummy variables are compared to zero*.

† *p < 0.1*,

** *p < 0.01*,

*** *p < 0.001*.

In both the IPD-MD and the positive variant of the IPD-MD, regardless of the order in which the games were played, groupness was negatively related to the number of tokens invested in the private account (i.e., groupness had a positive effect on overall contributions). When the IPD-MD was played first, groupness was positively related to both ingroup love and outgroup hate. In contrast, when the positive variant of the IPD-MD was played first, groupness was related—in both the initial positive variant and the subsequent IPD-MD—*only* to ingroup love, and not to outgroup hate.

## 4. Discussion

Previous research already established that when outgroup hate can be expressed by avoiding to help the outgroup and its members, discrimination is more likely to occur (Mummendey et al., 1992, 2000; Banaji and Greenwald, 2013; Greenwald and Pettigrew, 2014), and outgroup hate plays a more central role in the unfolding of intergroup conflict (Weisel and Böhm, 2015). The current works sheds further light on the interplay between ingroup love and outgroup hate by examining behavior of the same participants when outgroup hate can be displayed by harming the outgroup (IPD-MD) and by avoiding to help it (positive variant of the IPD-MD).

The analysis and interpretation in Weisel and Böhm (2015) assumed that the motivations in the IPD-MD and the positive variant of the IPD-MD are comparable, in the sense that contribution to the within-group account in each is motivationally similar to contribution to the between-group account in the other (and vice-versa). The results from the current study confirm this assumption (see Section 3.1).

More interesting, perhaps, are the different behavioral patterns between the IPD-MD and the positive variant of the IPD-MD. Behavior in the positive variant was rather stable; it did not depend on whether decisions were made before or after taking part in the IPD-MD. Behavior in the IPD-MD, however, was sensitive to the order. Interestingly, when the IPD-MD was played after a preceding positive variant, there was a very low level of outgroup hate (see Section 3.2). A possible explanation for this result is that the first situation (game) that people encounter establishes a set of available actions. In the positive variant of the IPD-MD this set of actions includes helping the ingroup only, or helping both the ingroup and outgroup, with outgroup hate being associated with the former. This association between outgroup hate and helping just the ingroup carries on to the subsequent IPD-MD, where helping just the ingroup is also an available action, such that even people with an initial inclination for outgroup hate opt for it.

This line of reasoning also accounts for the lack of a similar spillover effect when the IPD-MD was played first. In this case outgroup hate is initially associated with helping the ingroup and at the same time harming the outgroup. This combination, however, is not available in the subsequent positive variant, forcing participants to make a “fresh” choice. The result is that the level of outgroup hate is not affected by the preceding IPD-MD.

The order effect discussed above is not limited to displays of outgroup hate, but extends to the way peoples' sense of groupness relates to outgroup hate. When the IPD-MD was played first, i.e., when the first set of available actions participants were exposed to involved the possibility to harm the outgroup, groupness was related to ingroup love as well as to outgroup hate in both the initial IPD-MD and the subsequent positive variant of the IPD-MD (see Section 3.4). Strikingly, when the positive variant was played first, groupness was still related to ingroup love, but—in both the initial positive variant *and* the subsequent IPD-MD—*not* to outgroup hate. The negative effects of groupness can be avoided, it seems, if “positive” encounters take place first.

A straight forward implication of these results is that it is important that initial encounters between members of potentially conflicting groups take place in a positive context (e.g., student exchanges), where group members can have the option to help members of the other group, even if the future holds inevitable encounters in a negative context. In a similar vein, before deciding whether or not to join the army, perhaps it is better that young adults make a conscious decision about whether or not to volunteer for the red-cross, or for a similar organization that provides indiscriminate help. This can reduce conflict in subsequent encounters, even those where it is possible to harm the outgroup, and help harness group members' sense of groupness to constructive causes.

The reasoning above resonates well with research showing that awareness of harm imposed on an outgroup by members of the ingroup can increase the dehumanization of outgroup members (Castano and Giner-Sorolla, 2006; Čehajić et al., 2009), and that awareness of intergroup help can help rehumanize the outgroup (Saguy et al., 2015). The current results suggest that rather than awareness of actual intergroup harm or help that occurred in the past, negative and positive impressions of the outgroup, accompanied by the relevant motivational forces (outgroup hate/ingroup love), can arise by being in a situation where there is an opportunity to harm or to help the outgroup, even in the absence of information about the actual harming/helping behavior of other ingroup members.

Given the relatively low levels of outgroup hate they observed in the IPD-MD game, Halevy et al. (2008) assert that “intergroup conflicts can be resolved by channelling group members' altruism toward internal group causes” (p. 410). The current results suggest that if initial encounters between groups involve the opportunity to help the outgroup, or, possibly, if the encounters are framed such that the choice is between helping the outgroup or not, intergroup conflict can be reduced even further. Intergroup conflicts that involve the opportunity to harm or to help the outgroup not only evoke different motivations (e.g., Weisel and Böhm, 2015), but affect each other in different ways when they take place in succession.

## Funding

This work was supported by the Israel Science Foundation (grant 1392/08) and by the European Research Council (grant ERC-AdG 295707 COOPERATION).

### Conflict of interest statement

The author declares that the research was conducted in the absence of any commercial or financial relationships that could be construed as a potential conflict of interest.

## Footnotes

^1^Helping the ingroup and the outgroup might be considered as a display of *outgroup love*. Recall, however, that the two main motivations under consideration are ingroup love and outgroup hate.

^2^In the IPD-MD both the within-group account and the between-group account are discriminatory (albeit not to the same degree).

## References

[B1] Aaldering H. Greer L. L. Van Kleef G. A. De Dreu C. K. (2013). Interest (mis) alignments in representative negotiations: do pro-social agents fuel or reduce inter-group conflict? Organ. Behav. Hum. Decis. Process. 120, 240–250. 10.1016/j.obhdp.2012.06.00117689439

[B2] Allport G. W. (1954). The Nature of Prejudice. Reading, MA: Addison-Wesley.

[B3] Banaji M. R. Greenwald A. G. (2013). Blindspot: Hidden Biases of Good People. New York, NY: Delacorte Press.

[B4] Bates D. Maechler M. Bolker B. (2012). lme4: Linear Mixed-effects Models Using S4 Classes. R Package Version 0.999999-0.

[B5] Bornstein G. Ben-Yossef M. (1994). Cooperation in intergroup and single-group social dilemmas. J. Exp. Soc. Psychol. 30, 52–67. 10.1006/jesp.1994.1003

[B6] Brewer M. B. (1999). The psychology of prejudice: ingroup love and outgroup hate? J. Soc. Issues 55, 429–444. 10.1111/0022-4537.00126

[B7] Castano E. Giner-Sorolla R. (2006). Not quite human: infrahumanization in response to collective responsibility for intergroup killing. J. Pers. Soc. Psychol. 90:804. 10.1037/0022-3514.90.5.804 16737374

[B8] Čehajić S. Brown R. González R. (2009). What do i care? perceived ingroup responsibility and dehumanization as predictors of empathy felt for the victim group. Group Process. Intergroup Relat. 12, 715–729. 10.1177/1368430209347727

[B9] De Dreu C. K. Balliet D. Halevy N. (2014). Chapter one-parochial cooperation in humans: forms and functions of self-sacrifice in intergroup conflict. Adv. Motiv. Sci. 1, 1–47. 10.1016/bs.adms.2014.08.001

[B10] De Dreu C. K. W. Dussel D. B. Ten Velden F. S. (2015). In intergroup conflict, self-sacrifice is stronger among pro-social individuals, and parochial altruism emerges especially among cognitively taxed individuals. Front. Psychol. 6:572. 10.3389/fpsyg.2015.00572 25999888 PMC4422010

[B11] De Dreu C. K. W. (2010). Social value orientation moderates ingroup love but not outgroup hate in competitive intergroup conflict. Group Process. Intergroup Relat. 13, 701–713. 10.1177/1368430210377332

[B12] De Dreu C. K. W. (2012). Oxytocin modulates cooperation within and competition between groups: an integrative review and research agenda. Horm. Behav. 61, 419–428. 10.1016/j.yhbeh.2011.12.009 22227278

[B13] De Dreu C. K. W. Greer L. L. Handgraaf M. J. J. Shalvi S. Van Kleef G. A. Baas M.. (2010). The neuropeptide oxytocin regulates parochial altruism in intergroup conflict among humans. Science 328, 1408–1411. 10.1126/science.1189047 20538951

[B14] Greenwald A. G. Pettigrew T. F. (2014). With malice toward none and charity for some: ingroup favoritism enables discrimination. Am. Psychol. 69, 669–684. 10.1037/a0036056 24661244

[B15] Halevy N. Bornstein G. Sagiv L. (2008). “In-group love” and “out-group hate” as motives for individual participation in intergroup conflict: a new game paradigm. Psychol. Sci. 19, 405–411. 10.1111/j.1467-9280.2008.02100.x 18399895

[B16] Halevy N. Weisel O. Bornstein G. (2012). “In-group love” and “out-group hate” in repeated interaction between groups. J. Behav. Decis. Mak. 25, 188–195. 10.1002/bdm.726

[B17] Mummendey A. Otten S. Berger U. Kessler T. (2000). Positive-negative asymmetry in social discrimination: valence of evaluation and salience of categorization. Pers. Soc. Psychol. Bull. 26, 1258–1270. 10.1177/0146167200262007

[B18] Mummendey A. Simon B. Dietze C. Grünert M. Haeger G. Kessler S.. (1992). Categorization is not enough: intergroup discrimination in negative outcome allocation. J. Exp. Soc. Psychol. 28, 125–144. 10.1016/0022-1031(92)90035-I24072101

[B19] Pinheiro J. C. Bates D. M. (2000). Mixed-effects Models in S and S-PLUS. New York, NY: Springer Verlag.

[B20] R Core Team. (2012). R: A Language and Environment for Statistical Computing. Vienna: R Foundation for Statistical Computing.

[B21] Rusch H. (2014). The evolutionary interplay of intergroup conflict and altruism in humans: a review of parochial altruism theory and prospects for its extension. Proc. R. Soc. Lond. B Biol. Sci. 281:20141539. 10.1098/rspb.2014.1539 25253457 PMC4211448

[B22] Saguy T. Szekeres H. Nouri R. Goldenberg A. Doron G. Dovidio J. F. (2015). Awareness of intergroup help can rehumanize the out-group. Soc. Psychol. Pers. Sci. 6, 551–558. 10.1177/1948550615574748

[B23] Van Lange P. A. (1999). The pursuit of joint outcomes and equality in outcomes: an integrative model of social value orientation. J. Pers. Soc. Psychol. 77:337 10.1037/0022-3514.77.2.337

[B24] Weisel O. Böhm R. (2015). “Ingroup love” and “outgroup hate” in intergroup conflict between natural groups. J. Exp. Soc. Psychol. 60, 110–120. 10.1016/j.jesp.2015.04.008 26339099 PMC4518042

