# Supplementary material for: Negative and positive externalities in intergroup conflict: exposure to the opportunity to help the outgroup reduces the inclination to harm it
Source: Front Psychol. 2015 Oct 31;6:1594. doi: 10.3389/fpsyg.2015.01594 (PMC4628119; doi:10.3389/fpsyg.2015.01594)
Supplement: Supplementary file 1 [file Presentation1.PDF]

Supplementary Material: Negative and positive externalities in intergroup conflict: Exposure to the opportunity to help the outgroup reduces the inclination to harm it

Ori Weisel [1,*]

[1] *Centre for Decision Research and Experimental Economics, University of Nottingham, United Kingdom*

Correspondence*:
Ori Weisel
Centre for Decision Research and Experimental Economics, Unviersity of Nottingham, NG9 2RD, United Kingdom, ori.weisel@nottingham.ac.uk

1 SUPPLEMENTARY DATA

1.1 PAYOFF FUNCTIONS

Table 1. Payoff π_i of player i in group k. The number of tokens allocated by player i to the private, within-group, and between-group accounts, is denoted by p_i, w_i, b_i, respectively.

Game	N	Payoff function
IPD-MD	3	$\pi_i = 2 \times p_i + \sum_{j \in k}(w_j + b_j) - \sum_{j \notin k}(b_j)$
IPD-MD	6	$\pi_i = 2 \times p_i + 0.5 \times \sum_{j \in k}(w_j + b_j) - 0.5 \times \sum_{j \notin k}(b_j)$
Positive variant of the IPD-MD	3	$\pi_i = 2 \times p_i + \sum_{j \in k}(w_j + b_j) + \sum_{j \notin k}(b_j)$
Positive variant of the IPD-MD	6	$\pi_i = 2 \times p_i + 0.5 \times \sum_{j \in k}(w_j + b_j) + 0.5 \times \sum_{j \notin k}(b_j)$

2 SUPPLEMENTARY TABLES AND FIGURES

2.1 EFFECT OF GROUPNESS

The effect of groupness on contribution decisions was tested by means of generalized linear mixed effect models, using the lme4 package (Bates et al., 2012) in the R environment (R Core Team, 2012). Since each participant made two decisions, the specific participant was modelled as a random effect (Pinheiro and Bates, 2000). The explanatory variables were groupness, the game (dummy variable; IPD-MD (baseline) or positive variant of the IPD-MD), the position of the game (dummy variable; first (baseline) or second), and the two- and three-way interactions between these variables. The dependant variables were (in separate models) the number of tokens kept, contributed towards ingroup love (within-group pool in

IPD-MD, between-group pool in the positive variant of the IPD-MD), and contributed towards outgroup hate (between-group pool in IPD-MD, within-group pool in the positive variant of the IPD-MD).

The results are presented in Table 2. The significant and negative *Groupness* coefficient in the model predicting the number of tokens invested in the private account indicates that for the baseline values of the *Game* and *Played* dummies (i.e., IPD-MD played first), groupness was negatively related to the number of tokens invested in the private account. The lack of significant interaction effects suggests that this was the case regardless of the game (IPD-MD or the positive variant) and whether the game was played first or second.

A similar analysis with respect to the model predicting the number of tokens invested in ingroup love indicates a positive relation to groupness, which was again not sensitive to the game or the order.

The model predicting the number of tokens invested in outgroup hate shows a positive relation to groupness in the baseline, with a significant interaction between the *Groupness* and *Played* variables, indicating that the relation between groupness and outgroup hate was weaker for the game that was played second.

Table 2. Generalized linear mixed effects model. The number of tokens invested the private account, ingroup love, and outgroup hate (in separate models), as a function of groupness, the game (dummy variable with two levels: IPD-MD or the positive variant), whether the game was played first or second (dummy variable with two levels), and the interactions between these three variables.

	**Number of tokens invested in**		
	**private account**	**ingroup love**	**outgroup hate**
Intercept	11.21***	-0.83	-0.38
	(0.82)	(0.88)	(0.58)
Groupness	-1.19***	0.76***	0.43**
	(0.19)	(0.20)	(0.13)
**Game**			
IPD-MD (Ref)			
Positive variant of the IPD-MD	-0.17	-0.46	0.63
	(1.13)	(1.21)	(0.80)
**Position**			
First (Ref)			
Second	0.14	-0.67	0.53
	(1.13)	(1.21)	(0.80)
**Interactions**			
Groupness × Game(2)	-0.11	0.38	-0.27
	(0.25)	(0.27)	(0.18)
Groupness × Position (2)	-0.014	0.33	-0.35*
	(0.25)	(0.27)	(0.18)
Game(2) × Position(2)	-0.68	1.43	-0.75
	(1.93)	(2.12)	(1.34)
Groupness × Game(2) × Position(2)	0.17	-0.60	0.43
	(0.43)	(0.47)	(0.30)

Standard errors in parentheses; Ref = reference group.
The specific participant was considered as a random effect.
$^{\dagger}p < 0.1$, $*p < 0.05$, $**p < 0.01$, $***p < 0.001$

3 INSTRUCTIONS

The instructions in the following pages are translated from the Hebrew original. The instructions are for sessions with 3-persons group, where the IPD-MD (green world state) was played first, and the positive variant of the IPD-MD (yellow world state) was played second. Instructions for other types of sessions (6-person groups, other order of games) are nearly identical, with obvious differences.

The instructions for the game that was played second were only handed out after decisions in the first game were made.

[Translated from Hebrew]
[Introduction]

Experiment in decision making

In the experiment you will be asked to make decisions, and so will the other participants. Your decisions, and those made by the other participants, will determine the monetary payoff you will receive at the end of the experiment, according to rules that we will describe shortly.

You will be paid in cash at the end of the experiment exactly according to the rules.

Please remain quiet throughout the experiment. Do not communicate with the other participants. If you have a question, raise your hand, and one of the experimenters will come to your desk.

---

12 people are taking part in the experiment (11 in addition to you).

The 12 participants were randomly divided to four three-person groups (the circles group, the triangles group, the diamonds group, the squares group).

Each group is matched with another group.

You, and two other participants, are in the <u>circles</u> group.

Your group is matched with the <u>triangles</u> group.

The following diagram illustrates the division to groups and your group's matching:

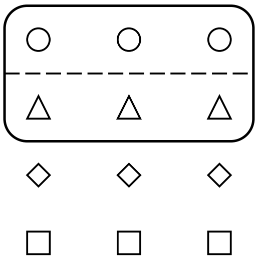

During the experiment you will make decisions in two world states, denoted by the colors yellow and green. At the end of the experiment one of the world states will be chosen randomly, by means of a public coin toss. The chosen world state will determine your monetary payoff.

In each of the world states you have a private account with an initial sum, as do the other participants. In addition, in each world state you will be required to make a decision that will determine, together with others' decisions, how many points will be added to, or subtracted from, your account, the account of your group members, and the accounts of the members of the other group.

The initial sum, and the rules that determine the payoffs, are different in the two world states.

The coin toss that will determine which world state determines the payoffs will take place at the end of the experiment, so consider each decision you make seriously, as if it is the one determining the payoffs.

[Translated from Hebrew]
[Instructions for green world state]
[Printed on green paper]

# - - - - - - - - - - - - - GREEN WORLD STATE - - - - - - - - - - - - -

Personal account

In this world state you, and each of the other participants, have an initial sum of 40 NIS in your private account.

Decision

Additionally, you and the other participants each have 10 tokens.

You (and each of the other participants) will have to decide how to invest the 10 tokens that you have.

- For each token that you keep for yourself, 2 NIS will be added to your private account.
- For each token you invest in pool A, your group will receive 3 tokens that will be divided equally among the group members (you, and each of your two group members, will receive 1 NIS to the private account).
- For each token you invest in pool B, your group will receive 3 tokens that will be divided equally among the group members, and the other group will lose 3 NIS, with the loss being equally divided among the group members (you, and each of your two group members, will receive 1 NIS to the private account, and 1 NIS will be subtracted from the private account of each of the three members in the other group).

[Translated from Hebrew]
[Instructions for green world state]
[Printed on green paper]

Example 1 – the effect of investment in the various pools on the payoff of participant X – Green world state

**Participant X**	Kept	4	tokens.	For each token X receives 2 NIS.	Total: 8 NIS
	Invested	3	tokens in pool A.	For each token X receives 1 NIS.	Total: 3 NIS
	Invested	3	Tokens in pool B.	For each token X receives 1 NIS.	Total: 3 NIS
**X's two group members:**	Kept	6	tokens.	No effect on X.	
	Invested	8	tokens in pool A.	For each token X receives 1 NIS.	Total: 8 NIS
	Invested	6	Tokens in pool B.	For each token X receives 1 NIS.	Total: 6 NIS
**Three members of the other group:**	Kept	10	tokens.	No effect on X.	
	Invested	11	tokens in pool A.	No effect on X.	
	Invested	9	Tokens in pool B.	For each token X loses 1 NIS.	Total: -9 NIS

Total payoff for participants X: 19 NIS

This amount will be **added** to the initial sum that is already in X's private account.

This is just an example. The actual payoff that you and the other group members will receive at the end of the experiment will be determined by the decisions you record on the official decision form.

[Translated from Hebrew]
[Instructions for green world state]
[Printed on green paper]

Here is another example of a possible outcome of the experiment.
To make sure you understand the rules, please fill in the blanks.
When you finish, raise your hand and an experimenter will come to your desk.

Example 2 – the effect of investment in the various pools on the payoff of participant X – Green world state

**Participant X**	Kept	3	tokens.	For each token X receives 2 NIS.	Total: ____
	Invested	4	tokens in pool A.	For each token X receives 1 NIS.	Total: ____
	Invested	3	Tokens in pool B.	For each token X receives 1 NIS.	Total: ____
**X's two group members:**	Kept	6	tokens.	No effect on X.	
	Invested	8	tokens in pool A.	For each token X receives 1 NIS.	Total: ____
	Invested	6	Tokens in pool B.	For each token X receives 1 NIS.	Total: ____
**Three members of the other group:**	Kept	10	tokens.	No effect on X.	
	Invested	9	tokens in pool A.	No effect on X.	
	Invested	11	Tokens in pool B.	For each token X loses 1 NIS.	Total: ____

Total payoff for participants X: 19 NIS

This amount will be **added** to the initial sum that is already in X's private account.

[Translated from Hebrew]
[Instructions for green world state]
[Printed on green paper]

- - - - - - - DECISION FORM - GREEN WORLD STATE - - - - - - -

Now you must decide how to invest your 10 tokens. Your decision, and the decisions of the other participants, will determine your payoff.

**For each token that you…**		
**Keep for yourself**<br>You receive 2 NIS<br><br>No effect of your group members<br><br>No effect on members of the other group	**Invest in pool A**<br>You receive 1 NIS<br><br>Each of your group members receives 1 NIS<br><br>No effect on members of the other group	**Invest in pool B**<br>You receive 1 NIS<br><br>Each of your group members receives 1 NIS<br><br>Each member of the other group loses 1 NIS

Draw a circle around your group, and write clearly how many tokens you wish to invest in each pool.

To be paid:

- You must mark the correct group (the one that you really belong to)
- The sum of tokens must equal 10

Draw a circle around your group

Circles Triangles Diamonds Squares

I wish to:

Keep _________ tokens for myself

Invest _________ tokens in pool A

Invest _________ tokens in pool B

[Translated from Hebrew]
[Instructions for yellow world state]
[Printed on yellow paper]

# - - - - - - - - - - - - - YELLOW WORLD STATE - - - - - - - - - - - - -

The yellow world state is similar to the green world state, but they are not identical. The differences are **highlighted**.

Personal account

In this world state you, and each of the other participants, have an initial sum of **10** NIS in your private account.

Decision

Additionally, you and the other participants each have 10 tokens.

You (and each of the other participants) will have to decide how to invest the 10 tokens that you have.

- For each token that you keep for yourself, 2 NIS will be added to your private account.
- For each token you invest in pool A, your group will receive 3 tokens that will be divided equally among the group members (you, and each of your two group members, will receive 1 NIS to the private account).
- For each token you invest in pool B, your group will receive 3 tokens that will be divided equally among the group members, and the other group will **receive** 3 NIS that will be divided equally among the group members (you, and each of your two group members, will receive 1 NIS to the private account, and 1 NIS will be **added** to the private account of each of the three members in the other group).

[Translated from Hebrew]
[Instructions for yellow world state]
[Printed on yellow paper]

Example 1 – the effect of investment in the various pools on the payoff of participant X – Green world state

**Participant X**	Kept	4	tokens.	For each token X receives 2 NIS.	Total: 8 NIS
	Invested	3	tokens in pool A.	For each token X receives 1 NIS.	Total: 3 NIS
	Invested	3	Tokens in pool B.	For each token X receives 1 NIS.	Total: 3 NIS
**X's two group members:**	Kept	6	tokens.	No effect on X.	
	Invested	8	tokens in pool A.	For each token X receives 1 NIS.	Total: 8 NIS
	Invested	6	Tokens in pool B.	For each token X receives 1 NIS.	Total: 6 NIS
**Three members of the other group:**	Kept	10	tokens.	No effect on X.	
	Invested	11	tokens in pool A.	No effect on X.	
	Invested	9	Tokens in pool B.	For each token X receives 1 NIS.	Total: 9 NIS

Total payoff for participants X: 37 NIS

This amount will be **added** to the initial sum that is already in X's private account.

This is just an example. The actual payoff that you and the other group members will receive at the end of the experiment will be determined by the decisions you record on the official decision form.

[Translated from Hebrew]
[Instructions for yellow world state]
[Printed on yellow paper]

Here is another example of a possible outcome of the experiment.
To make sure you understand the rules, please fill in the blanks.
When you finish, raise your hand and an experimenter will come to your desk.

Example 2 – the effect of investment in the various pools on the payoff of participant X – Green world state

**Participant X**	Kept	5	tokens.	For each token X receives 2 NIS.	Total: ____
	Invested	3	tokens in pool A.	For each token X receives 1 NIS.	Total: ____
	Invested	2	Tokens in pool B.	For each token X receives 1 NIS.	Total: ____
**X's two group members:**	Kept	7	tokens.	No effect on X.	
	Invested	6	tokens in pool A.	For each token X receives 1 NIS.	Total: ____
	Invested	7	Tokens in pool B.	For each token X receives 1 NIS.	Total: ____
**Three members of the other group:**	Kept	11	tokens.	No effect on X.	
	Invested	7	tokens in pool A.	No effect on X.	
	Invested	12	Tokens in pool B.	For each token X receives 1 NIS.	Total: ____

Total payoff for participants X: ____

This amount will be **added** to the initial sum that is already in X's private account.

[Translated from Hebrew]
[Instructions for yellow world state]
[Printed on yellow paper]

- - - - - - DECISION FORM - YELLOW WORLD STATE - - - - - -

Now you must decide how to invest your 10 tokens. Your decision, and the decisions of the other participants, will determine your payoff

**For each token that you…**		
**Keep for yourself**	**Invest in pool A**	**Invest in pool B**
You receive 2 NIS<br><br>No effect of your group members<br><br>No effect on members of the other group	You receive 1 NIS<br><br>Each of your group members receives 1 NIS<br><br>No effect on members of the other group	You receive 1 NIS<br><br>Each of your group members receives 1 NIS<br><br>Each member of the other group receives 1 NIS

Draw a circle around your group, and write clearly how many tokens you wish to invest in each pool.

To be paid:

- You must mark the correct group (the one that you really belong to)
- The sum of tokens must equal 10

Draw a circle around your group

Circles Triangles Diamonds Squares

I wish to:

Keep _________ tokens for myself

Invest _________ tokens in pool A

Invest _________ tokens in pool B

REFERENCES

Bates, D., Maechler, M., and Bolker, B. (2012). lme4: Linear mixed-effects models using S4 classes. R package version 0.999999-0.

Pinheiro, J. C. and Bates, D. M. (2000). *Mixed-effects models in S and S-PLUS*. Springer Verlag, New York.

R Core Team (2012). *R: A Language and Environment for Statistical Computing*. Vienna, Austria. ISBN 3-900051-07-0.
